# Initial Pro Re Nata Brolucizumab for Exudative AMD: The PROBE Study

**DOI:** 10.3390/jcm10184153

**Published:** 2021-09-15

**Authors:** Alper Bilgic, Laurent Kodjikian, Samaresh Srivastava, Shyamal Dwivedi, Alay S Banker, Amro Abukashabah, Aditya Sudhalkar, Thibaud Mathis

**Affiliations:** 1 Alphavision Augenarztpraxis Clinic, 27568 Bremerhaven, Germany; drbilgicalper@yahoo.com; 2 Service d’Ophtalmologie, Centre Hospitalier Universitaire de la Croix-Rousse, Hospices Civils de Lyon, Université Claude Bernard Lyon 1, 69004 Lyon, France; laurent.kodjikian@chu-lyon.fr (L.K.); dr.heartaaa@hotmail.com (A.A.); thibaud.mathis@chu-lyon.fr (T.M.); 3 UMR-CNRS 5510 Laboratory, Matéis, Villeurbane, 69100 Lyon, France; 4 Raghudeep Eye Hospital, Ahmedabad 380054, India; samaresh@raghudeepeyeclinic.com (S.S.); shyamal@raghudeepeyeclinic.com (S.D.); 5 Banker Retina Clinic, Ahmedabad 380054, India; alay.banker@gmail.com; 6 Ophthalmology Department, King Abdulaziz University, Rabigh 25732, Saudi Arabia; 7 MS Sudhalkar Medical Research Foundation, Baroda 390001, India

**Keywords:** age-related macular degeneration, anti-vascular endothelial growth factor, brolucizumab, exudation, treatment-naive

## Abstract

The present study aimed to determine the efficacy and safety of pro re nata (PRN) intravitreal brolucizumab therapy for neovascular age-related macular degeneration (AMD) without a loading dose in the real-world setting. The PROBE study (Pro Re Nata Brolucizumab for Exudative AMD) is a retrospective, observational, multicentric study that included 27 treatment-naïve patients (27 eyes) with neovascular AMD who received PRN brolucizumab therapy with the treatment interval being at least 8 weeks, should the need for a second consecutive injection arise. The primary outcome measure was changed to best-corrected visual acuity (BCVA) over time. Secondary outcome measures included the determination of change in central subfield thickness (CST) and complications. The mean follow-up was 11.2 ± 1.2 months. The mean baseline and final BCVA were 57.4 ± 4.5 letters and 65.3 ± 3.12 letters, respectively (*p* = 0.014). The mean gain in letters at the end of follow-up was 7.8 ± 3.5 letters. There was a significant decrease in CST at the end of the follow-up period (*p* = 0.013). Patients received a mean of 2.2 ± 0.9 injections (in addition to the first mandatory injection) during the follow-up period. There were no adverse events noted. In conclusion, initial PRN brolucizumab for exudative AMD without a loading dose demonstrated significant visual improvement and no adverse events.

## 1. Introduction

Age-related macular degeneration (AMD) is the leading cause of blindness in the elderly in industrialized countries. There are two forms of the disease—atrophic or neovascular—the latter being characterized by the formation of new blood vessels either under or above the retinal pigment epithelium (RPE). Before the advent of anti-vascular endothelial growth factor (VEGF) therapy, thermal laser, intravitreal steroid injections, and photodynamic therapy, or a combination of these, were considered the standard of care. Anti-VEGF agents have revolutionized therapy for neovascular age-related macular degeneration (nAMD) [1,2]. Although these molecules provide excellent results when injected every month, visual loss is observed when the treatment is given less frequently [3]. A decade’s experience of anti-VEGF therapy has taught us to minimize therapy and to maximize visual gains, thereby sparing patients the physical and psychological burden of multiple treatment visits [4] and the potential threat of geographic atrophy (although this rarely manifests, if ever) [5]. Pro re nata injections and other less frequent injection protocols [6,7,8,9,10] attempt to achieve this without compromise on visual outcomes. An alternative approach would be to look at more potent and durable formulations that obviate the need for intense therapy.

The latest development in anti-VEGF therapy has been the introduction of brolucizumab, a 26 kDa anti-VEGF antibody that is far smaller than currently available agents such as ranibizumab, bevacizumab, or aflibercept. This allows the manufacturer to pack a higher molecular concentration into the standard 0.05 mL volume, in the hope of increasing the durability of the molecule in the intravitreal compartment. The HAWK and HARRIER studies have established the non-inferiority of the new molecule brolucizumab compared to aflibercept, with some analyses suggesting a superior anatomic outcome [11]. Nearly 50% of enrolled patients could receive 12 weekly injections, considerably reducing the treatment burden. However, concerns about safety with special reference to intraocular inflammation and vasculitis have dampened the initial enthusiasm for the drug [12]. As the data evolves, the risk of serious adverse events is continuously updated (www.brolucimab.info, accessed on 29 May 2021) [13]. The reported predisposing factors for intraocular inflammation following brolucizumab injection include female gender, multiple past treatments, and frequent injections, among others [14].

The current analysis investigated the efficacy and safety of pro re nata brolucizumab for nAMD in a real-world setting. 

## 2. Materials and Methods

The PROBE (Pro Re Nata Brolucizumab for Exudative AMD) study is an observational, retrospective, multicenter study conducted at the Sudhalkar and Raghhudeep group of hospitals in India. A database search was performed for patients with treatment-naïve macular neovascularization (MNV) who received brolucizumab as intravitreal therapy. This study complied with the tenets of the Declaration of Helsinski and was approved by the ethics committee for the Raghudeep Eye Hospital, Ahmedabad, India. Patients provided informed consent for participation in the study.

### 2.1. Eligibility

The PROBE study examined the outcomes in treatment-naïve patients with nAMD who received PRN intravitreal brolucizumab therapy. Patients needed to complete a minimum of 10 months follow-up for inclusion. Patients with polypoidal choroidal vasculopathy (PCV) or retinal angiomatous proliferation (RAP) were excluded.

### 2.2. Definitions and Grading

Type I MNV (historically called ‘occult’ neovascularization) was defined by the presence of a neovascular membrane under the RPE layer. Type II MNV (historically called ‘classic’ neovascularization) was defined by the presence of a neovascular membrane above the RPE layer. A mixed lesion was defined by the presence of both neovascular components: type 1 and type 2 MNV. Macular fluid was classified as intraretinal (IRF) or subretinal fluid (SRF) according to the recent consensus guidelines [15]. Fluid disappearance post-injection was considered to be a complete response. Pigment epithelial detachment (PED) was noted if present, but it was not considered to be an independent treatment criterion as in the HAWK and HARRIER trials. A recurrence was defined as a complete resolution of fluid in the intraretinal and/or subretinal compartment followed by recurrent fluid in at least one compartment. Baseline images were graded independently by two of the investigators (AS and AB) and adjudicated by a senior colleague (LK). Patients received one mandatory injection at baseline; subsequent injections were administered only if persistent fluid was present more than 8 weeks after the first injection. Even if there was persistent fluid at the end of 4 weeks, patients were followed up until 8 weeks.

### 2.3. Acquisition of Data

Data retrieved included patient demographics; the best-corrected visual acuity as recorded using the Early Treatment Diabetic Retinopathy Study (ETDRS) chart (also mentioned in the manuscript in Snellen’s notations for ease of interpretation); the best-corrected visual acuity (BCVA); intraocular pressure (IOP); the details of the ocular examination and special investigations conducted, such as fluorescein angiography (FA) and/or indocyanine angiography (ICGA) and central subfield thickness (CST) as determined by SD-OCT (Heidelberg Spectralis, Heidelberg Engineering, Heidelberg, Germany); the type of MNV (type 1/type 2/mixed); the size and location thereof; the anti-VEGF agents used; the number of injections administered; the treatment-free interval; and a switch to an alternative anti-VEGF agent, if any. In addition, BCVA, measurement of IOP, slit-lamp examination, fundoscopy, and SD-OCT were documented at each visit.

### 2.4. Follow-Up

Intravitreal injections were performed using a standardized aseptic technique. Follow-up was performed on days 1, 7, 15, and 30 following the first injection, and was then followed monthly. SD-OCT scans were performed at weeks 2, 4, and 8, and then every 4 weeks.

### 2.5. Outcome Measures

The primary outcome measure was taken to determine the change in BCVA from baseline with treatment. Secondary outcome measures included the change of CST in SD-OCT, the mean number of injections required to achieve the complete resolution of exudation, and any complications associated therewith. 

### 2.6. Statistical Analysis

This being a real-world study, the number of eyes recruited for analysis was based on past literature that looked at less frequent therapy without compromise on visual outcomes [16]. The description of categorical variables was based on absolute (size) and relative (percentage) frequencies. Quantitative variables were represented as the mean and standard deviation. The comparison of the categorical variables between the groups of different indications was performed using Fisher’s exact test. When the pairwise comparisons were subsequently performed, the *p*-value was adjusted using the Benjamini–Hochberg method, wherever applicable. A *p*-value < 0.05 was considered to be statistically significant.

## 3. Results

A total of 27 eyes of 27 patients (15 females and 12 males) have received PRN intravitreal brolucizumab at our centers thus far and have completed at least 10 months of follow-up. Table 1 lists the salient characteristics of these eyes, and this analysis forms the basis for our study.

The mean time to treatment after the beginning of symptoms was 37.2 ± 11.5 days. The baseline BCVA was 57.4 ± 4.5 letters and the mean CST was 398.1 ± 47.2 µm. The most frequent MNV was type 1, and the mean area of the neovascular membrane was 169.4 ± 34.5 µm.

**Figure 1:**
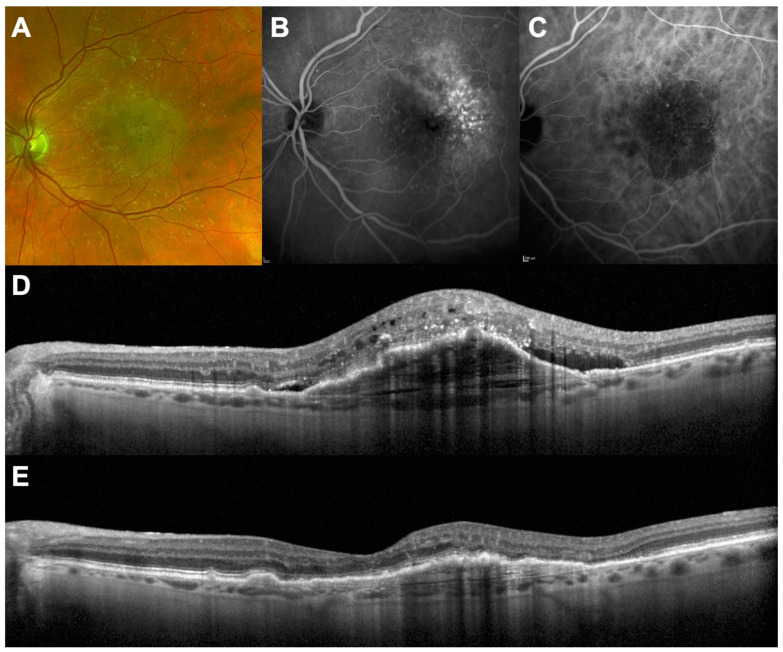
A 72-year-old female with vision loss and metamorphopsia in her left eye beginning 5 days previously. (**A**–**C**): Multimodal imaging at baseline showing type 1 macular neovascularization in a context of exudative AMD; (**D**) SD-OCT showing pigment epithelial detachment, subretinal fluid, and intraretinal fluid. Visual acuity was 68 letters; (**E**) SD-OCT 1 month after a single intravitreal injection of brolucizumab showing total regression of retinal fluid. Visual acuity increased to 76 letters.

A total of 7/27 eyes (25.9%) showed completely resolved exudation after one injection, 13/27 eyes (48.2%) showed complete resolution of exudation after two injections and the remaining seven eyes (25.9%) needed three or more injections (Figure 1). Recurrence in exudation was seen in 23/27 eyes (85.2%) prior to the end of follow-up. Recurrence was seen a mean of 3.7 ± 1.2 months after the last injection. Four eyes (14.8%) did not show any recurrence in exudation prior to the last follow-up.

The mean CST decreased significantly to 283.0 ± 57.2 µm at the final visit from the presentation CST of 398.1 ± 47.2 µm (*p* = 0.021). Patients received an average of 2.2 ± 0.9 brolucizumab injections (range: 1–4 injections in addition to the first mandatory injection) over the mean course of 11.2 ± 1.2 months.

### 3.1. Visual Gain

**Figure 2:**
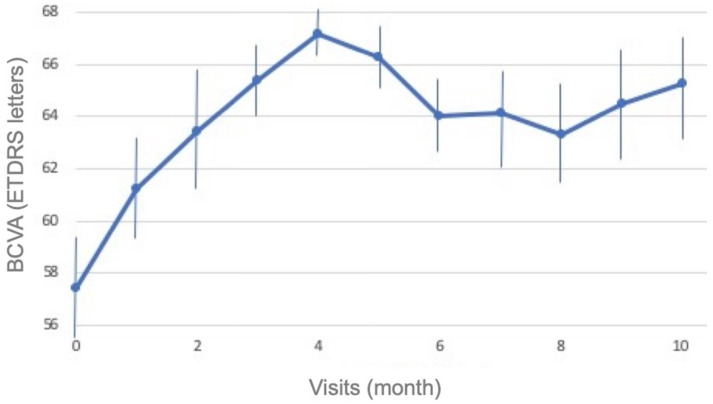
Evolution of best-corrected visual acuity (BCVA) during the follow-up period.

The mean BCVA significantly increased from the baseline (57.4 ± 4.5 letters) to the final visit (65.3 ± 3.1 letters; *p* = 0.014). The mean letter gain in vision was 7.8 ± 3.5 letters (Figure 2). A total of 5/27 eyes (18.5%) gained 15 letters or more from baseline at one month after the loading dose and another 7/27 eyes (25.9%) showed a 10-letter gain. At the end of the follow-up, 14/27 patients (51.9%) retained a BCVA ≥ 20/30. Moreover, none of the patients lost letters from the baseline. 

### 3.2. Adverse Events

We did not note a single case of intraocular inflammation during follow-up in any of the 27 eyes. We also did not note any instance of post-injection endophthalmitis or visual loss in any of the study eyes until the last follow-up. None of the patients was required to be switched to an alternative anti-VEGF agent.

## 4. Discussion

The present study showed good functional and anatomical results following the intravitreal injections of brolucizumab in a PRN regimen for the treatment of naïve eyes with nAMD. Moreover, almost a half of patients demonstrated a significant vision gain of ≥10 to 15 letters, occurring as early as 1 month after the first injection. Approximately three quarters of eyes demonstrated complete resolution of exudation with two injections, and a quarter showed complete resolution with one injection. The chosen minimum retreatment interval of 8 weeks was based on the findings of the HAWK and HARRIER studies, which showed a low rate of disease activity of patients treated with brolucizumab [11]. No significant adverse events were reported here. Despite the longer injection interval, our visual gains are readily comparable to the findings of the HAWK and HARRIER studies. This demonstrates that the chosen treatment interval may not adversely influence the visual gain while fulfilling our anatomical objectives of a dry macula. In addition to our study, other real-life studies have demonstrated the effectiveness of brolucizumab injections in naïve, but also switch patients [17,18,19,20,21]. These data, combined with the present study, reinforce the results of the pivotal randomized controlled trial [11].

Since the advent of anti-VEGF therapy, numerous studies have demonstrated that monthly injections provide high and sustained visual gain, but were found to be rather impractical and perhaps even unsustainable in the long term. Past literature is replete with instances of different studies looking at less intensive treatment regimens. The PRN regime is one such protocol, as is the treat-and-extend strategy. Some studies have even questioned the need for a loading dose. Monés et al. have looked at the possibility of combining fixed interval and PRN injections for nAMD, thereby reducing the total number of requisite treatments while maintaining visual acuity gains comparable to historical evidence published for monthly injections [16]. Moreover, it has also been shown that a single dose followed by a PRN strategy provided comparable and sustained visual gains to strategies that incorporated a loading dose followed by PRN therapy [22]. Finally, we recently showed that some patients need only one anti-VEGF injection over the long term, arguing against the historical three loading doses [23]. In the same way, the treat-and-extend regimen was introduced as an alternative to monthly injections, allowing adjustment of the reinjection interval by 2 weeks according to the disease activity. Recently, the ALTAIR study looked at liberalization of the treatment regimen by introducing four weekly extensions to the treatment interval as opposed to the standard two weekly extensions [24]. 

Brolucizumab was designed to provide better efficacy and a longer duration of action, thereby promoting a longer treatment interval. It follows, then, that every new anti-VEGF molecule that is approved for use in wet AMD should have its own treatment protocol and that past experience and older protocols with similar molecules may guide present protocols but may not always be replicated. In other words, monthly injections or treat-and-extend protocols may actually be considered obsolete as far as brolucizumab is concerned; this is evolving data, however, and only long-term analyses will point towards more appropriate treatment regimens. Moreover, the original concept of loading dose should be revisited, in our opinion, given that it was formulated for molecules (such as ranibizumab and aflibercept) that were far less potent and had a far lower molecular concentration than brolucizumab. The results found herein support this new paradigm of treatment as we demonstrated good outcomes provided by a strategy based on one initial injection of brolucizumab followed immediately by PRN injections. This can be explained by the small size of the molecule, which allows a higher concentration of brolucizumab to be delivered to the vitreous in comparison to other FDA-approved drugs. The large dose of anti-VEGF probably accounts for the improved efficacy and durability, despite a higher rate of hypersensitivity-like reactions that are rarely reported for other agents [25]. The initial enthusiasm for this new molecule has been somewhat offset by reports of an increased propensity to produce inflammatory side effects, such as hyalitis or vasculitis [14,17,26]. However, due to the limited therapeutic arsenal available in the treatment of nAMD, brolucizumab could be an effective alternative anti-VEGF molecule. One explanation for the increased incidence of inflammatory events could probably be the increased ocular concentration of the drug and thereby its degradation products. It has already been hypothesized that it is these degradation products that lead to trabecular meshwork clogging [27] and thus a sustained rise in intraocular pressure. It is probable that this accumulation of degradation products also influences a currently poorly understood inflammatory reaction. However, this needs further analysis. It is with this phenomenon under consideration that we decided to explore PRN brolucizumab for nAMD.

The main limitation of the present study was its retrospective design and relatively small size. Some data may be missing, and some patients may have been lost to follow-up. It is possible that our study was not sufficiently powered to determine the incidence of intraocular inflammation (currently reported to be around 5%, increasing to approximately 9% in the MERLIN trial, NCT03710564). However, we only included patients with a minimum of 10 months follow-up, thus providing useful information on the first year of treatment. Although the eyes included were fewer than in most trials, the follow-up was adequate. Intraocular inflammation has been observed, overall, after a mean of two injections in most eyes and is more common amongst females. In addition, real-life observational studies allow the analysis of populations with characteristics that are different from those included in randomized studies, such as those with high or low baseline visual acuity. Finally, the aim of this study was to provide a foothold for future alternatives as well as to open up newer avenues for treatment regimens that may signify a break from traditional monthly or treat-and-extend protocols. Indeed, monthly injections are probably an important reason for the high number of instances of intraocular inflammation noted in the recently aborted MERLIN study (NCT03710564). Finally, the loading dose concept need not necessarily entail four weekly injections. For molecules with a longer duration of action, even an eight weekly schedule should be equally effective. This requires further analysis.

To conclude, intravitreal brolucizumab therapy is effective when administered PRN in treatment-naïve patients with nAMD. We did not note any adverse events during the follow-up. Furthermore, the extended treatment interval did not compromise visual gains when compared to historically published literature. The initial PRN regimen with brolucizumab for nAMD thus appears to be a valid alternative.

## Figures and Tables

**Table 1:** Baseline characteristics of patients with treatment-naïve nAMD who received brolucizumab therapy.

Characteristic	Treatment Naïve (*N* = 27)
Mean age, years (SD)	65.1 (3.4)
Male:Female, *n*	12:15
Follow-up, months (SD)	11.2 (1.2)
Mean BCVA, letters (SD)	57.4 (4.5)
Mean CST, µm (SD)	398.1 (47.2)
MNV subtype, *n*:	
Type I	16
Type II	8
Mixed	3
Fluid localization, *n* *:	
IRF	18
SRF	8
PED	16

BCVA: best-corrected visual acuity; CST: central subfield thickness; IRF: intraretinal fluid; MNV: choroidal neovascularization; PED: pigment epithelium detachment; SD: standard deviation; SRF: subretinal fluid. * Patients could have fluid in more than one compartment.

## Data Availability

All data are available upon request to the corresponding author.
